# Calcitriol-mediated hypercalcemia as an immune-related adverse event in a patient receiving nivolumab and ipilimumab for metastatic renal cell carcinoma, case report

**DOI:** 10.1186/s12894-021-00825-4

**Published:** 2021-04-01

**Authors:** Kai Johnson, Majd Issa, Anish Parikh, Paul Monk, Ming Yin, Amir Mortazavi, Yuanquan Yang

**Affiliations:** Medical Oncology, Ohio State University James Cancer Hospital, Suite 1335 Lincoln Tower, 1800 Cannon Drive, Columbus, OH 43210 USA

**Keywords:** Autoimmunity, Case report, Immunotherapy, Kidney neoplasms, Urologic neoplasms

## Abstract

**Background:**

Severe hypercalcemia is often associated with uncontrolled malignancy through several mechanisms. However, calcitriol-mediated hypercalcemia is a rare etiology for advanced solid tumors.

**Case presentation:**

We report a case of calcitriol-mediated hypercalcemia secondary to immune checkpoint inhibition in a responder with metastatic clear cell renal cell carcinoma (ccRCC). In this case, a 68 year old male with metastatic ccRCC to the liver within 4 months of right radical nephrectomy went on to develop hypercalcemia (12.8 mg/dL) shortly following 2 cycles of nivolumab and ipilimumab. Additional testing showed an elevated calcitriol level (142 pg/mL), low parathyroid hormone (PTH) and parathyroid hormone-related protein (PTHrP) levels, and a normal 25-hydroxyvitamin D level. FDG-PET imaging showed hypermetabolic mediastinal, hilar, and intra-abdominal lymphadenopathy, however the subsequent lymph node biopsy only showed reactive lymphoid cells without malignancy or granuloma. The hypercalcemia was resistant to initial therapy with calcitonin, hydration, and zoledronic acid but quickly responded to high-dose prednisone (1 mg/kg), followed by normalization of calcitriol levels. The patient was rechallenged with nivolumab and ipilimumab which provided a partial response after 4 cycles. He was maintained on low dose prednisone (10 mg daily) leading to a sustained resolution of his hypercalcemia.

**Conclusion:**

This case suggests calcitriol-mediated hypercalcemia as a novel immune-related adverse event.

## Background

In the setting of malignancy, multiple mechanisms have been found to cause hypercalcemia. The most common of these are local osteolysis due to bony invasion from solid and hematologic malignancies (e.g., breast cancer), humoral hypercalcemia of malignancy due to paraneoplastic hormones (e.g., PTHrP), ectopic hyperparathyroidism, and calcitriol-secreting tumors (e.g., lymphoma) [1, 2]. Calcitriol-mediated hypercalcemia accounts for approximately 1% of cases of hypercalcemia in the setting of malignancy [3]. Under normal physiologic circumstances, vitamin D is stored in the liver as 25-hydroxyvitamin D. When hypocalcemic, it is released due to the presence of PTH before ultimately undergoing conversion to calcitriol by renal 1-α-hydroxylase. Calcitriol then signals for both increased calcium absorption via the intestines, as well as increased osteolytic activity in the bone. The cumulative effect leads to improved serum calcium levels. Certain malignancies are able to produce extra-renal 1-alpha-hydroxylase, leading to increased production of calcitriol. This is true most commonly with lymphoid malignancies, especially in the setting of progressive disease, whereas with solid tumors this is a rare occurrence [4]. In such rare cases involving solid tumors, alternative mechanisms for explaining calcitriol-mediated hypercalcemia must be explored, particularly when therapies themselves may play a role, such as in the case described below.

## Case presentation

The case involves a 68 year old male with metastatic clear cell renal cell carcinoma (ccRCC). His medical history was significant for well-controlled hypertension, stage 3 chronic kidney disease, and psoriatic arthritis which was controlled with hydroxychloroquine maintenance. He was referred to our clinic after presenting to a local provider with acute gross hematuria. He underwent a CT urogram that revealed a 7.3 cm mass in the right kidney with renal vein invasion and small, non-specific pulmonary nodules in the bilateral lung bases. He underwent a right radical nephrectomy within a month of his initial diagnosis. Pathology revealed a 6 cm (pT3a pNx) WHO grade 4 renal cell carcinoma, clear cell type. The tumor extended to the resection margin of the renal vein. Adjuvant radiation was not recommended at that time and he was placed on surveillance. Restaging scans four months after surgery showed stable sub-centimeter pulmonary nodules and multiple new hypodense hepatic lesions, the largest being 5.7 × 4.4 cm involving the central right hepatic lobe. No bone metastases were identified on imaging. A liver biopsy confirmed the diagnosis of metastatic ccRCC. He was then initiated on nivolumab 3 mg/kg and ipilimumab 1 mg/kg shortly afterwards. He tolerated the first cycle well but did develop a grade 1 rash involving his chest within a week of the infusion. This resolved with use of topical steroids. Two days following his cycle 2 infusion, he began to develop arthralgias that were attributed to a flare of his known psoriatic arthritis. On day 7 of cycle 2, he noticed progressive fatigue and nausea. On day 14 of cycle 2, he had an episode of emesis and was brought back to our clinic given his progression of symptoms. Routine labs at the time revealed new hypercalcemia to 12.8 mg/dL (previously 9.6 mg/dL two weeks prior) and that he had developed an acute kidney injury (creatinine 3.62 mg/dL, up from baseline of 1.6 to 1.9 mg/dL). He was admitted to our inpatient service for further testing, part of which is summarized in Table 1.

**Table 1:** Summary of lab tests performed and their respective values along with reference ranges

Lab test	Lab value
1, 25-dihydroxyvitamin D3 (calcitriol)	142 pg/ml (reference 20–79 pg/ml)
25-hydroxyvitamin D (25{OH}D)	36.7 ng/mL (reference 30–100 ng/mL)
Phosphorous	5.4 mg/dL (reference 2.2–4.6 mg/dL)
Parathyroid hormone (PTH)	< 6.3 pg/mL (reference 14.0–72.0 pg/mL)
Parathyroid hormone-related peptide (PTHrP)	0.7 pmol/L (reference ≤ 4.2 pmol/L)
Adrenocorticotropic hormone (ACTH)	273.0 pg/mL (reference 9.0–50.0 pg/mL)
Cortisol	37.03 mcg/dL (reference 3.09–22.40 mcg/dL)
Angiotensin-converting enzyme level	62 ng/dL (reference 9–67 ng/dL)
Alkaline phosphatase	110 U/L (reference 32–126 U/L)

**Fig. 1:**
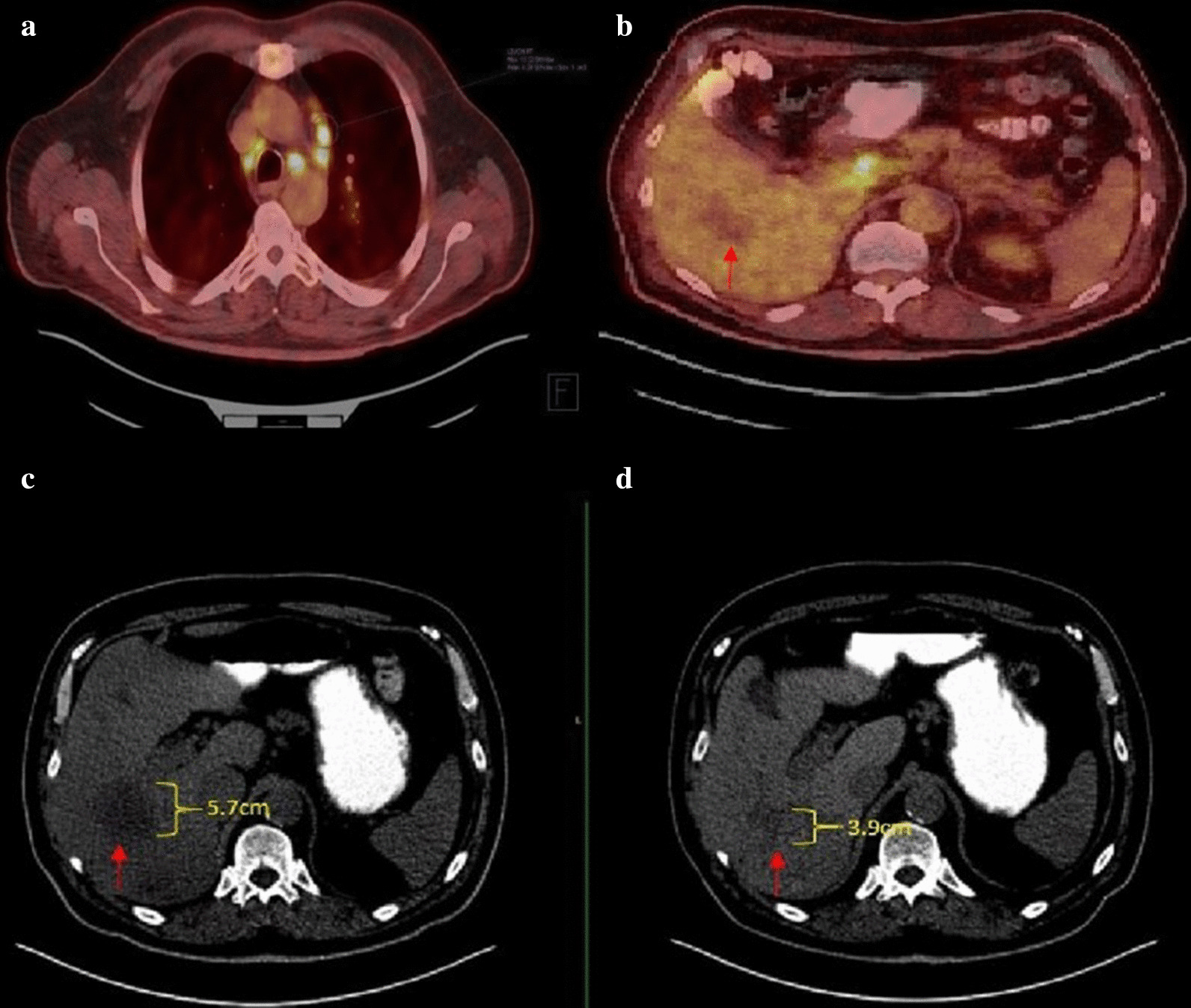
**a** FDG-PET/CT demonstrating hypermetabolic mediastinal lymph nodes. **b** FDG-PET/CT showing a hypermetabolic portacaval lymph node, though the known liver metastasis was not FDG-avid (red arrow). Below these PET images, two CT scans of the abdomen & pelvis are shown: one before treatment was initiated (**c**) and one 6 months after treatment was completed (**d**). The dominant right liver lesion decreased from 5.7 × 4.4 cm to 3.9 × 3.6 cm in size (red arrow)

**Fig. 2:**
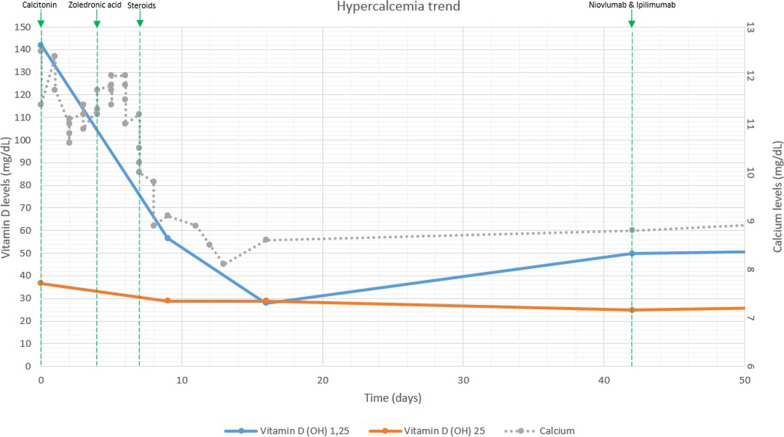
This graph demonstrates the changes in levels of calcium (shown in gray), 1, 25(OH) D (shown in blue), and 25(OH) D (shown in orange) throughout the patient’s hospitalization as various treatments were provided as labeled above. The X axis represents time measured in days. The left Y axis illustrates vitamin D levels in mg/dL. The right Y axis demonstrates the calcium levels in mg/dL. The green dashed lines indicate the start dates for the various treatments described above including steroids (prednisone), zoledronic acid, calcitonin, and immunotherapy (nivolumab and ipilimumab re-challenge)

His inpatient workup was significant for an elevated 1, 25-dihydroxyvitamin D3 (calcitriol) level of 142 pg/mL (reference 20–79 pg/mL) in the presence of low-normal 25-hydroxyvitamin D (25{OH} D) at 36.7 ng/mL (reference 30–100 ng/mL), suggesting alpha hydroxylation. Other hypercalcemia testing revealed an elevated phosphorus level of 5.4 mg/dL (reference 2.2–4.6 mg/dL), a suppressed parathyroid hormone (PTH) of less than 6.3 pg/mL (reference 14.0–72.0 pg/mL), a low parathyroid hormone-related peptide (PTHrP) of 0.7 pmol/L (reference ≤ 4.2 pmol/L), an elevated adrenocorticotropic hormone (ACTH) level of 273.0 pg/mL (reference 9.0–50.0 pg/mL), an elevated cortisol level of 37.03 mcg/dL (reference 3.09–22.40 mcg/dL), a normal alkaline phosphatase level of 110 U/L (reference 32–126 U/L), and a normal angiotensin-converting enzyme level of 62 ng/dL (reference 9–67 ng/dL). FDG-PET imaging showed multiple hypermetabolic mediastinal, hilar and intra-abdominal lymph nodes (max SUV of 15), as illustrated in Fig. 1. The liver lesions themselves *were* not FDG-avid but were stable in size, as were his prior lung nodules. He underwent a bronchoscopy with biopsy of four separate lymph nodes, none of which demonstrated malignancy nor granulomatous disease. He was initially treated with intravenous normal saline, calcitonin 4 units/kg every 12 h and 4 mg zoledronic acid. Despite seven days of aggressive treatment, his hypercalcemia persisted, as shown in Fig. 2. He was then started on 1 mg/kg of oral prednisone both for this calcitriol-mediated hypercalcemia as well as for his ongoing psoriatic arthritis flare. Within two days of starting systemic steroids, the patient’s calcitriol level normalized (56.6 pg/mL), as did his calcium level (8.9 mg/dL). He reported notable relief of arthritic symptoms immediately. He was then discharged home and slowly weaned down to prednisone 10 mg daily, which was continued for control of his arthritis.

As an outpatient, he was re-challenged with nivolumab and ipilimumab 1 month later. Restaging CT scans after cycle 4 revealed an overall partial response, with the largest liver lesion shrinking by 32% in the long-axis (3.6 × 3.9 cm from 4.4 × 5.7 cm), as seen in Fig. 1. Prior to receiving cycle 6 nivolumab maintenance, routine lab work showed the development of immunotherapy-related hypothyroidism for which thyroid hormone replacement therapy was started. He remains on nivolumab maintenance therapy with ongoing partial response of his disease, no recurrent hypercalcemia, and no further immunotherapy-related adverse events (irAE) to date.

## Discussion and conclusions

We report the first-known case of calcitriol-mediated hypercalcemia associated with immunotherapy. We come to this etiologic conclusion given the laboratory findings of a suppressed PTH, low PTHrP, and an elevated calcitriol level. In addition, multiple PET-avid mediastinal lymph nodes were biopsied and failed to demonstrate the presence of malignancy or granulomatous disease, though this does not definitively rule out either condition. Our suspicion that this may be an irAE is based on multiple factors, including onset soon after immunotherapy initiation, the concurrent worsening of his chronic autoimmune disorder (psoriatic arthritis), the rapid normalization of calcitriol levels with the introduction of high-dose glucocorticoid therapy, and partial responses noted on restaging scans. Given these findings, it seems likely that this episode of calcitriol-mediated hypercalcemia was in relation to immune checkpoint blockade.

Despite this likely association, the mechanism of dysregulated calcitriol production in the setting of immunotherapy is unclear. It is well-known that certain inflammatory conditions with granuloma formation, such as sarcoidosis, can present with calcitriol-induced hypercalcemia [5]. This process is mediated through the abnormal activation of macrophages, which play an important role in calcium homeostasis. They are an extra-renal source of calcitriol production through stimulation of their own unique 25(OH) D-hydroxylase enzyme, which is insensitive to PTH and is less susceptible to negative inhibition through its substrate as compared to its renal counterpart [6–8]. A subset of macrophages from cancer patients also express PD-1 [9]. The blockade of PD-1/PD-L1 with monoclonal antibodies can activate macrophages, increase phagocytosis activity and reduce tumor growth. It is possible that the calcitriol-mediated hypercalcemia of our patient was due to macrophage activation by nivolumab, an anti-PD-1 antibody. Furthermore, it is well-established that PD-1/PD-L1 blockade activates T cells. Activated T cells express an increased level of 25(OH) D-hydroxylase [10]. The local conversion of 25(OH) vitamin D to 1, 25(OH)_2_ vitamin D is important for T cell function [11]. Corticosteroids help block hydroxylase enzymes responsible for this vitamin D conversion as well as inhibit osteoclast resorption through suppression of tumor cytokine production [12].

In conclusion, we report a rare case of calcitriol-mediated hypercalcemia as a novel immunotherapy-related adverse event in the absence of granulomas. Steroids played a central role in the management of this case. Additional study is needed to further characterize calcitriol production in response to immunotherapy within various immune cell types in vitro and the role of vitamin D supplementation in cancer immunotherapy.

## Data Availability

Available medical records along with an extensive literature search were used to report these findings. The data used and analyzed during the current study are available from the corresponding author on reasonable request.

## Abbreviations

ccRCC: Clear cell renal cell carcinoma
PTH: Parathyroid hormone
PTHrP: Parathyroid hormone-related peptide
ACTH: Adrenocorticotropic hormone
SUV: Standardized uptake values
irAE: Immunotherapy-related adverse events
